# Presentation pattern and survival rate of retinoblastoma following chemotherapy: a prospective study

**DOI:** 10.1186/s12887-023-04347-w

**Published:** 2023-10-28

**Authors:** Mayor Orezime Atima, Ugbede Idakwo, Oyeronke Komolafe, Shimizu Eisuke, Nakayama Shintaro, Emmanuel Oluwadare Balogun, Emeka John Dingwoke, Ayodele Jacob Orugun, Kenneth Onosigho Ukumobe, Jah Douglas Pam, Amaka Aladiuba

**Affiliations:** 1 ECWA Eye Hospital, Kano, Nigeria; 2 Department of Ophthalmology, Keio University School of Medicine, Tokyo, Japan; 3 Department of Biochemistry, Faculty of Life Sciences, Ahmadu Bello University, Zaria, Kaduna State Nigeria

**Keywords:** Retinoblastoma, Presentation, Pediatrics, ECWA eye hospital, Nigeria

## Abstract

**Background:**

This study presents the clinical pattern of presentation and survival rate of retinoblastoma, which is the most prevalent form of pediatric intraocular cancer. The aim of this study is to provide baseline information about the clinical presentation and management of retinoblastoma at ECWA Eye Hospital. Additionally, the study identifies priority areas for enhancing medical care for children diagnosed with this cancer. ECWA Eye Hospital, situated in Kano State, Nigeria, is a specialized eye center located in the North-Western region of the country.

**Methods:**

A prospective study spanning five years was conducted at ECWA Eye Hospital to investigate clinically diagnosed cases of retinoblastoma. The study took place from January 2018 to December 2022. The patients received standardized pre-medication and chemotherapy protocols for retinoblastoma. Subsequently, a five-year follow-up was conducted to monitor the patients’ progress. The collected data was analyzed, descriptive statistics were generated, and the survival rate was calculated.

**Results:**

During the five-year study period, a total of 35 cases of retinoblastoma were diagnosed. The patients had an average age of 3.21 ± 1.32 years. The most common presentation patterns observed were fungating ocular mass and proptosis. Among the cases, there were 10 instances of bilateral proptosis and 25 instances of unilateral proptosis. While no patients exhibited bilateral leukocoria, eight cases of unilateral leukocoria with anterior segment seedlings were identified. The additional patterns of presentation are proptosis, leukocoria, fungating orbital mass, redness and loss of vision. The mortality rate was 80% (28 cases), while the survival rate was 20% (7 cases). Notably, all the survivors had unilateral retinoblastoma.

**Conclusion:**

The majority of cases observed at ECWA Eye Hospital involve advanced retinoblastoma. In low-resource settings where alternative treatment options are limited, chemotherapy is considered a viable treatment option. Early presentation of retinoblastoma in patients may lead to a higher survival rate when chemotherapy is administered.

## Introduction

Retinoblastoma (RB) is a prevalent form of pediatric cancer that originates from the embryonal cells of the retina. It is the most commonly occurring primary intraocular cancer in children and has a high likelihood of being successfully treated, particularly if detected in its early stages [1, 2]. Cancer can be attributed to two genetic events that impact both alleles of the *RB1* gene, which acts as a tumor suppressor. This condition can occur in either a hereditary or non-hereditary manner [3]. The hereditary form is caused by germline mutations in the *RB1* gene and is commonly observed to manifest bilaterally [4, 5]. The non-hereditary variant of RB is characterized by somatic mutations in the RB1 gene and typically presents unilaterally [4, 5]. Individuals with the hereditary form of RB are at a lifelong risk of developing a second primary tumor [2]. The most frequently observed symptom during initial presentation is leukocoria, while other signs of RB may include squint, impaired vision, redness, or proptosis [6, 7].

More than twenty years ago, developed nations achieved remarkable survival rates in cases of RB, focusing their therapeutic efforts on preserving the eye and visual function [8]. In contrast, developing countries, including Nigeria, continue to experience a high mortality rate associated with RB [7, 9, 10]. The majority of cases in Nigeria are characterized by unilateral disease, followed by bilateral and trilateral diseases [11, 12]. Poor survival rates in RB have been associated with factors such as late presentation, lack of awareness, poor compliance, and inadequate healthcare facilities [13–15]. In the United States, the occurrence of RB is approximately 1 in 15,000–20,000 live births, and 60% of patients are diagnosed with unilateral disease [4]. Globally, it is estimated that around 9,000 cases are diagnosed each year, with a higher annual prevalence observed in Africa compared to higher-income countries [10, 16].

The Evangelical Church Winning All (ECWA) eye hospital in Nigeria is a dedicated missionary and specialized tertiary eye care facility that provides treatment for a diverse range of eye disorders. The aim of this study was to examine the clinical characteristics, patterns of presentation, and survival outcomes of patients diagnosed with RB. More so, to establish fundamental knowledge regarding the clinical presentation and treatment of RB while identifying key areas for enhancing medical care for children affected by this form of cancer.

## Materials and methods

### Ethical consideration, inclusion and exclusion criteria

This study received approval from the human research ethics committee of ECWA Eye Hospital in 2017, with the approval number ECWA/EH/003/2017, and strictly adhered to the standards set forth in the Helsinki Declaration. Informed consent was duly obtained from the participants as required. It was registered at https://www.researchregistry.com, with the Research Registry Unique Identifying Number: 9593.

### Inclusion criteria

Patients who provided consent for the study and successfully completed six courses of chemotherapy, as well as those who were unable to be reached but still completed the full six courses of chemotherapy, and those who had histological confirmation of retinoblastoma, were all included in the study.

### Exclusion criteria

Patients who lacked histological confirmation of the disease, declined to have their eyes enucleated as recommended, and did not provide their consent were excluded from the study.

### Procedures

This is a five-year follow-up prospective study conducted on 35 pediatric patients who were treated for retinoblastoma at the ECWA Eye Hospital Kano. The study involved a comprehensive evaluation of all suspected cases of retinoblastoma by an Ophthalmologist, which included a detailed medical history, physical examination, and specialized diagnostic tests. Patients who received a diagnosis of retinoblastoma underwent further evaluation by a Pediatrician and subsequently underwent an examination under anesthesia (EUA). Chemotherapy was administered to patients with confirmed retinoblastoma. The staging of the tumor was determined using the Intraocular Retinoblastoma Classification (IIRC) [17].

Patients scheduled for chemotherapy received pre-medications consisting of intravenous infusion of 4.3% dextrose saline (Pediatric saline: PS) 500ml every 12 h for a duration of 24 h. Additionally, they were administered a bolus of Dexamethasone at a dosage of 0.25 mg/kg, and closely monitored for any adverse reactions for a period of 15 min. Subsequently, Ondansetron at a dosage of 0.15 mg/kg, in 100ml of PS, was administered intravenously over a period of 30 min. Patients were then observed for an additional 15 min.

Following the initial procedure, Carboplatin was administered at a dosage of 28 mg/kg in 250 ml of PS over a duration of 1 h. This was then followed by a 1-hour observation period under 250 ml of 4.3% PS. Subsequently, Etoposide was administered at a dosage of 12 mg/kg in 250 ml of PS, which ran for 1 h. Another hour of observation under 250 ml of PS followed this. Finally, a bolus of IV Vincristine was given at a dosage of 0.05 mg/kg. Patients were monitored for 24 h for any adverse reactions before being discharged.

This treatment cycle consisting of chemotherapy was repeated a total of six times, with each cycle being spaced 28 days apart. Due to delayed presentation, the maximum dose of chemotherapy was administered. Enucleation surgery was performed after the second or third dose of chemotherapy, depending on the patient’s suitability for the procedure. Patients were followed up for a period of five years, undergoing EUA twice a year, with all findings meticulously documented in their medical records. Two of the patients experienced recurrences during the follow-up period and were subsequently given two additional courses of chemotherapy. The survival rate was determined by the absence of recurrence in patients who completed all six courses of chemotherapy during the five-year follow-up period.

### Statistical analysis

The data was evaluated descriptively using Statistical Package for Social Sciences (SPSS) version 21. The results were presented as the mean ± standard deviation.

## Results

In our healthcare facility, a total of 39 patients diagnosed with retinoblastoma were treated and monitored during the study period, four patients were lost to follow-up. Among the remaining 35 patients who received comprehensive management and follow-up, there were 18 males and 17 females, with an average age of 3.21 ± 1.32 years. The distribution of patients according to gender and age is summarized in Table 1. Specifically, there were 10 cases of bilateral retinoblastoma and 25 cases of unilateral retinoblastoma.

**Table 1:** Retinoblastoma distribution frequency in patients

Gender	Age (Yrs.)	Frequency	Bilateral	Unilateral
Male	3.21 ± 1.32	18	4	14
Female		17	6	11
**Total**		**35**	**10**	**25**

The presentation pattern is illustrated in Table 2. Among the cases observed, there were 10 instances of bilateral proptosis, with 4 occurring in males and 6 in females. Additionally, there were 17 cases of unilateral proptosis, with 8 occurring in males and 9 in females. No cases of bilateral leukocoria with anterior segment seedlings were found, but 8 cases of unilateral leukocoria with anterior segment seedlings were identified, consisting of 6 males and 2 females.

**Table 2:** Laterality and Pattern of presentation of retinoblastoma patients

Gender	Proptosis		Leukocoria with anterior segment seedlings	
	Bilateral	Unilateral	Bilateral	Unilateral
**Male**	4	8	0	6
**Female**	6	9	0	2
Total	10	17	0	8

**Fig. 1:**
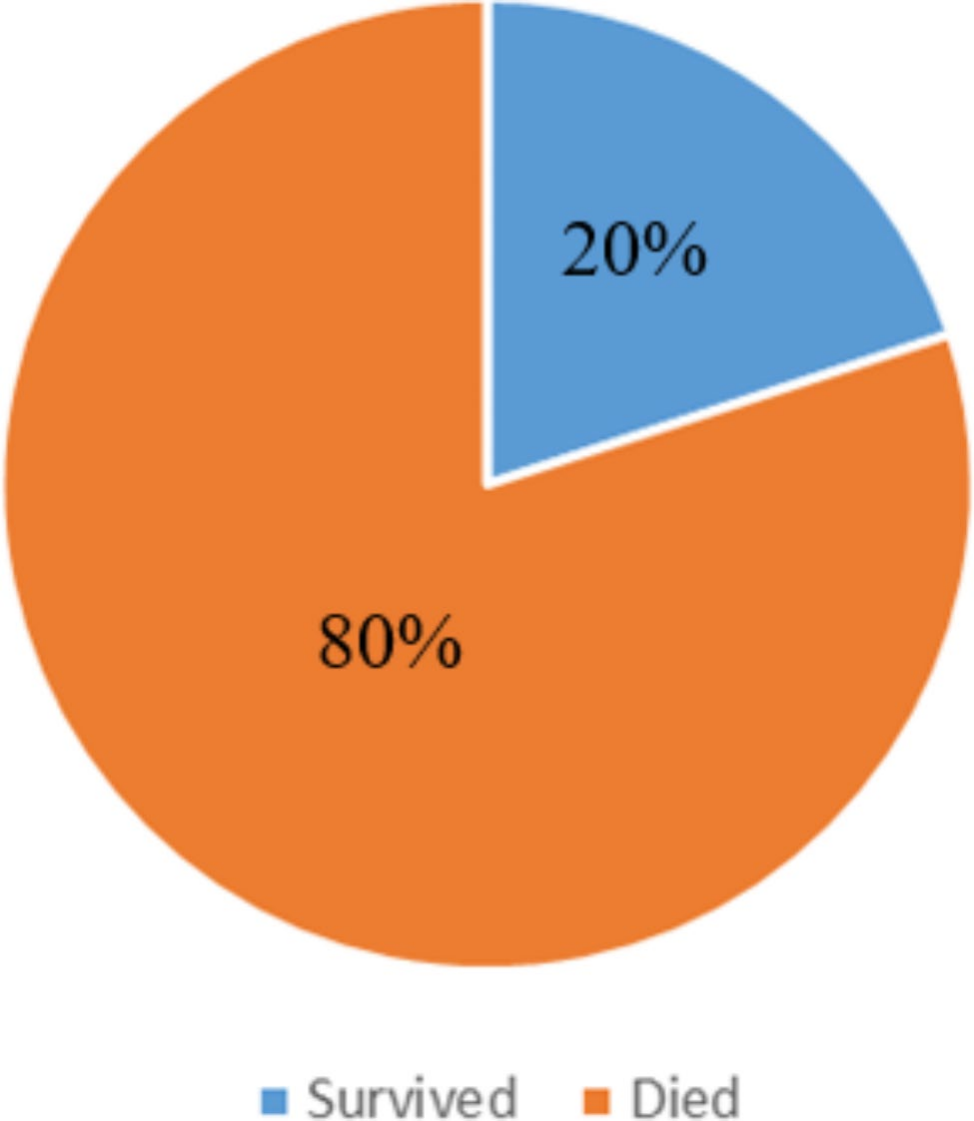
Survival and mortality after Five years

The data regarding survival and mortality over a five-year follow-up period is presented in Fig. 1. The survival rate was 20%, with a total of 7 patients who survived, while the mortality rate was 80%, accounting for 28 patients. The individuals who survived had unilateral retinoblastoma and exhibited symptoms of leukocoria and anterior segment seedlings.

The subcategories of retinoblastoma cases and their corresponding outcomes are summarized in Table 3. Out of the 35 patients, 28 (80%) had distant spread to the head and auricular lymph nodes, while only 7 (20%) had ocular involvement at presentation. All patients with distant metastasis had died within five years of follow-up, whereas those with intraocular tumors survived at the end of the five-year follow-up. Notably, all patients (100%) with distant metastasis had died by the end of the five-year follow-up period, while 100% of those with intraocular tumors but no optic nerve involvement had survived. Late presentation was observed in 28 (80%) of the 35 patients, resulting in death before the completion of the five-year period. Conversely, only 7 (20%) who presented early were still alive at the end of the five-year follow-up, as indicated in Table 3.

**Table 3:** Subcategories of retinoblastoma cases, presentation and survival rates

	Bilateral proptosis	Unilateral proptosis		Leukocoria	
**Type of spread of presentation**	Distant metastasis to the head and auricular Lymph node	Distant metastasis to the head and auricular Lymph node	Orbital spread auricular Lymph node	Orbital spread and pre auricular lymph node	Ocular spread only
**Survival rate (at 5 years) of the spread at presentation**	0%	0%	0%	0%	100%
**Period of presentation**	Late with distant metastasis	Late with distant metastasis	Late with distant metastasis	Late with distant metastasis	Early
**Survival rate (at 5 years) of presentation**	0%	0%	0%	0%	100%
**Total**	10	14	3	1	7

## Discussion

This study examined the presentation pattern and survival rates of retinoblastoma at a specialized eye facility in the Northwest region of Nigeria. Our findings align with previous research indicating that retinoblastoma is the most prevalent tumor observed in children [18–20]. The cases of retinoblastoma observed at our center had an average age of 3.21 ± 1.32 years, with both genders represented. The age range observed in our study (Table 1) is similar to certain regions within Nigeria, but differs from others. For instance, a study in the South-east [21] reported an age range of 5 months to 6 years, while a study in Lagos reported a range of 24.4 ± 11.4 months [22]. A mean age of 33.68 ± 12.27 months was reported in a study from the South-South region [23]. Other Sub-Saharan African countries reported 3 years in a combined study from the Republic of Côte d’Ivoire and the Democratic Republic of the Congo [20], while Egypt reported a range of 2-49 months [24].

One noteworthy aspect of the study is that it has provided valuable information regarding the prevalence of retinoblastoma cases in the North-west region of Nigeria. It is worth mentioning that the estimated number of retinoblastoma patients in various Sub-Saharan African areas is largely unknown [12]. This prospective analysis spanning five years revealed a total of 17 cases of unilateral proptosis and 10 cases of bilateral proptosis (Table 2). The most commonly observed symptoms upon presentation were fungating orbital mass, proptosis, leukocoria with seedling in the anterior segment and painful red eyes, as indicated in Table 3. It has been established that early diagnosis plays a crucial role in successfully treating this primary intraocular cancer in children [1, 2]. Unfortunately, late clinic presentation remains a significant obstacle, as evidenced by previous studies [11, 20–22]. Our study revealed a survival rate of merely 20%, indicating that 80% of the patients unfortunately did not survive the disease (Fig. 1; Table 3). While developed countries have witnessed substantial improvements in the survival rate of children with retinoblastoma [25, 26], the prognosis remains poor in developing countries. This is primarily due to delayed diagnosis and treatment discontinuation or refusal of enucleation, which are prevalent risk factors compromising the overall survival rate in countries where retinoblastoma is prevalent [10, 27]. Our findings are consistent with a study conducted in India that investigated the survival and outcomes of retinoblastoma patients who underwent neoadjuvant chemotherapy. The study reported notable mortality rates [26], which were attributed to the late-stage diagnosis of the disease during presentation and an increased susceptibility to both local and distant metastases [26].

**Fig. 2:**
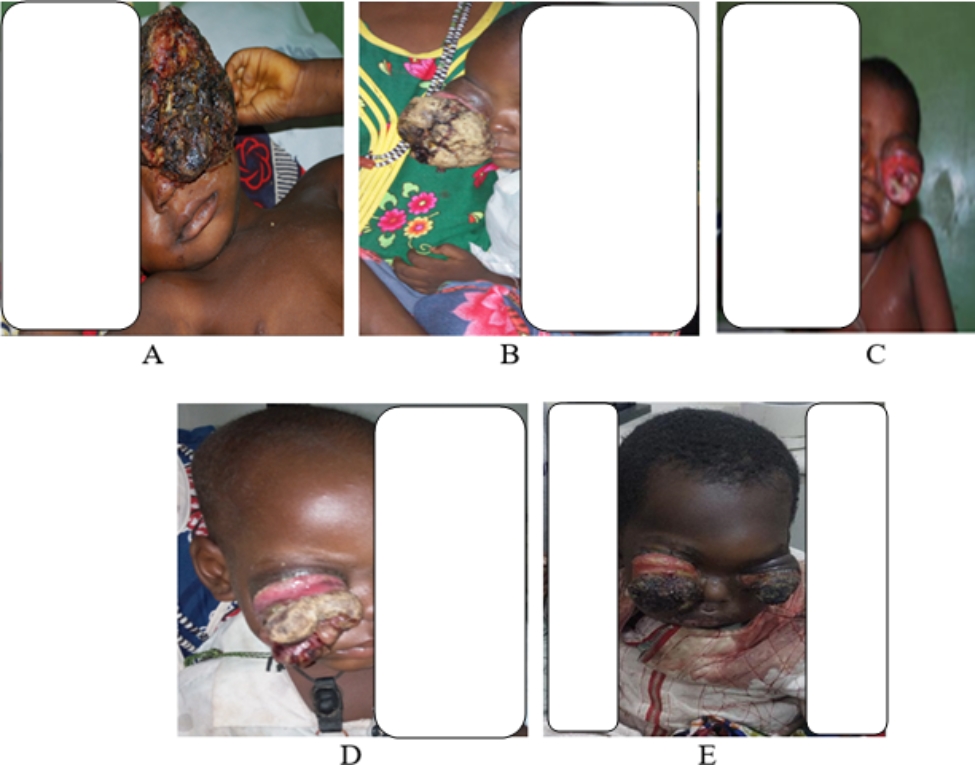
Photographs of the retinoblastoma cases seen in our center **A**: Unilateral LE, **B**: Unilateral RE, **C**: Unilateral LE, **D**: Unilateral RE, **E**: Bilateral retinoblastoma (RE – right eye, LE –Left eye)

It has been noted that the persistent lack of awareness regarding childhood eye diseases, specifically retinoblastoma, can result in delayed clinic visits [11, 28]. Childhood vision screening is widely recognized as a crucial method for identifying vision abnormalities and cancers like retinoblastoma in children [29, 30]. Based on our own experience, the primary symptom that prompts presentation and referral is proptosis (Fig. 2). In our center, 10 cases of bilateral proptosis and 17 cases of unilateral proptosis were observed (Table 2). These findings are consistent with the patterns observed in other studies, suggesting that the most common modes of presentation at retinoblastoma centers in Nigeria are extraocular extension of retinoblastoma and proptosis [11, 21, 22].

One of the limitations of this study is the small sample size, which can be attributed to various factors such as a lack of awareness about retinoblastoma, fear of undergoing eye removal, uncertainty regarding where to seek treatment, financial constraints, and reliance on traditional healers. The four patients who were lost to follow-up were likely unable to continue due to unfortunate circumstances such as death, financial difficulties, or insecurity in Nigeria, which led them to migrate to inaccessible areas. To address the challenges posed by limited resources in developing countries, it is crucial to establish a state-of-the-art center that is on par with those found in developed countries. Additionally, an intensive primary eye care information and health education campaign should be implemented, with a specific focus on promoting child eye health and early detection of retinoblastoma.

## Conclusion

Advanced retinoblastoma comprises the majority of cases observed at ECWA Eye Hospital, with the prevailing presentation pattern being a protruding ocular mass accompanied by fungating growth. At this stage, the disease exhibits extraocular dissemination and associated complications, leading to unfavorable survival outcomes. Proptosis, Leukocoria, Fungal Orbital Mass, Redness and Visual Loss are common presentation patterns. In resource-limited settings where alternative treatment modalities are not readily available, chemotherapy is a viable therapeutic approach. Early presentation of retinoblastoma patients, coupled with chemotherapy, can potentially enhance survival rates.

## Data Availability

The dataset generated during this study is available upon reasonable request from the corresponding author, Mayor Orezime Atima (atimamatha@yahoo.com).
